# Probing genetic control of swine responses to PRRSV infection: current progress of the PRRS host genetics consortium

**DOI:** 10.1186/1753-6561-5-S4-S30

**Published:** 2011-06-03

**Authors:** Joan K Lunney, Juan Pedro Steibel, James M Reecy, Eric Fritz, Max F Rothschild, Maureen Kerrigan, B Trible, Raymond RR Rowland

**Affiliations:** 1 Animal Parasitic Diseases Laboratory, BARC, ARS, USDA, Beltsville, MD 20705, USA; 2 Depts. Animal Science, and Fisheries and Wildlife, Michigan State Univ., East Lansing, MI 48824, USA; 3 Dept. Animal Science, Center for Integrated Animal Genomics, Iowa State Univ., Ames, IA 50011, USA; 4 Dept. Diagnostic Medicine and Pathobiology, College of Veterinary Medicine, Kansas State Univ., Manhattan, KS 66506, USA

## Abstract

**Background:**

Understanding the role of host genetics in resistance to porcine reproductive and respiratory syndrome virus (PRRSV) infection, and the effects of PRRS on pig health and related growth, are goals of the PRRS Host Genetics Consortium (PHGC).

**Methods:**

The project uses a nursery pig model to assess pig resistance/susceptibility to primary PRRSV infection. To date, 6 groups of 200 crossbred pigs from high health farms were donated by commercial sources. After acclimation, the pigs were infected with PRRSV in a biosecure facility and followed for 42 days post infection (dpi). Blood samples were collected at 0, 4, 7, 10, 14, 21, 28, 35 and 42 dpi for serum and whole blood RNA gene expression analyses; weekly weights were recorded for growth traits. All data have been entered into the PHGC relational database. Genomic DNAs from all PHGC1-6 pigs were prepared and genotyped with the Porcine SNP60 SNPchip.

**Results:**

Results have affirmed that all challenged pigs become PRRSV infected with peak viremia being observed between 4-21 dpi. Multivariate statistical analyses of viral load and weight data have identified PHGC pigs in different virus/weight categories. Sera are now being compared for factors involved in recovery from infection, including speed of response and levels of immune cytokines. Genome-wide association studies (GWAS) are underway to identify genes and chromosomal locations that identify PRRS resistant/susceptible pigs and pigs able to maintain growth while infected with PRRSV.

**Conclusions:**

Overall, the PHGC project will enable researchers to discover and verify important genotypes and phenotypes that predict resistance/susceptibility to PRRSV infection. The availability of PHGC samples provides a unique opportunity to continue to develop deeper phenotypes on every PRRSV infected pig.

## Introduction

Porcine reproductive and respiratory syndrome virus (PRRSV), first identified in the United States in 1987, costs U.S. swine producers >$560 million annually [1]. PRRSV-infected pigs are susceptible to pneumonia and growth losses; infected sows have increased rates of abortions, stillbirths, mummifications, and give birth to weak piglets with chronic respiratory problems. It can take weeks, even months, for pigs to clear this RNA virus, which evolves and adapts quickly to new environmental challenges such as vaccines and medications [2]. Reports of highly virulent PRRSV variants that have spread throughout China and into Vietnam highlight the importance of developing effective interventions to prevent PRRSV pathology, mortality and production losses [3-5].

Research has indicated that there are genetic components involved in determining how effective each pig will be in responding to and clearing PRRSV infection [6-17]. As discussed during the 2010 Animal Genomics for Animal Health International Symposium deep phenotypes are required for identifying the genes and pathways that are responsible for genetic control of PRRSV infection responses. Large numbers of animals and a resulting broad sample set is required to probe the numerous parameters involved. Such an effort requires that several entities pool their funding and scientific resources. In the US, the PRRS Host Genetics Consortium (PHGC) has been developed to coordinate a PRRS host genetics and resistance project. The objectives for the PHGC are to: 1) Use genotyping and phenotyping tools to determine if there are host genes that control resistance/susceptibility to PRRSV infection; 2) Verify genetic variation in response to PRRSV, via improved health, survivability and growth; and 3) Identify relative importance of different phenotypic traits, and their heritability, that predict response to PRRSV infection.

## Methods

### PHGC Planning

A large project such as the PHGC requires careful planning and commitments from diverse groups. The PHGC was developed at three one-day US National Pork Board meetings (15/12/05; 23/2/06; 9/5/07) with further input from PRRS CAP and NC229 disease researchers, NC1037/NRSP8 genome researchers, members of the NPB Swine Health and Animal Science Committees, veterinarians and the American Association of Swine Veterinarians, producers, and commercial partners representing breeders, animal health, feed, and diagnostic companies. The final plan is a result of those detailed discussions.

### Pig sources

All tests have been performed on commercial pigs from high health farms with donation of swine genetic materials as part of the Consortium. For each trial one company was requested to provide 200 pigs at weaning from PRRSV negative (PRRSV-), *Mycoplasma hyopneumoniae*-, and swine influenza virus- farms, and if possible from porcine circovirus type 2 (PCV2) free farms. Pigs could be from vaccinated sows since maternal antibody prevents them from becoming infected with PCV2. The source populations were crossbred commercial pigs (Genus/PIC USA; Newsham Choice Genetics; Fast Genetics; Genetiporc, Inc.; Genesus Genetics) with complete parentage and pedigree records. Since SNP chips are used for genotyping there was a decreased need for extensive family structure. There was no preselection of sires for disease traits. Pigs (~200/trial) were transported to the biosecure Kansas State University (KSU) testing facility at weaning. All pig experiments were initiated after approval by KSU IACUC and IBC institutional committees. After arrival pigs were treated with broad spectrum antibiotics for 1 week, to prevent expression of other organisms.

### Infection and phenotypic tests

After the 7 day acclimation period, pigs were challenged intranasally/orally with PRRSV isolate NVSL 97-7985 [18] and followed for 42 days post infection (dpi). Blood samples are collected at -6, 0, 4, 7, 10, 14, 21, 28, 35, 42 dpi for a total of 10 bleeds. Both sera and ABI Tempus tubes for later RNA analyses were collected at all sampling times. All samples were aliquoted and stored at KSU and BARC. Pigs were weighed weekly for growth data. Pigs were killed at 42 dpi and tonsils collected for viral persistence and ears for genomic DNA. If there are pig deaths during the study, dead pigs are sent for full workup at the KSU Diagnostic Lab.

### Consortium database

The Consortium relational database http://www.animalgenome.org/lunney/index.php is the secure data repository for all pig data including parentage information, location and availability of all samples collected on each pig, results of all assays performed on each sample (phenotypic and genotypic information). The database resides on computers located at Iowa State University, which are supported by NRSP8 Bioinformatics funds. All data collected through the project will be available to project members prior to publication and then to general public after original publication. Access to samples and to accumulated data stored in the secure PHGC Database is open to members who contribute materials or data. [Access to PHGC Database is monitored by the USDA ARS maintained Cooperative Research and Development Agreement (CRADA) Material Transfer Agreement (MTA).]

## Results and discussion

**Figure 1:**
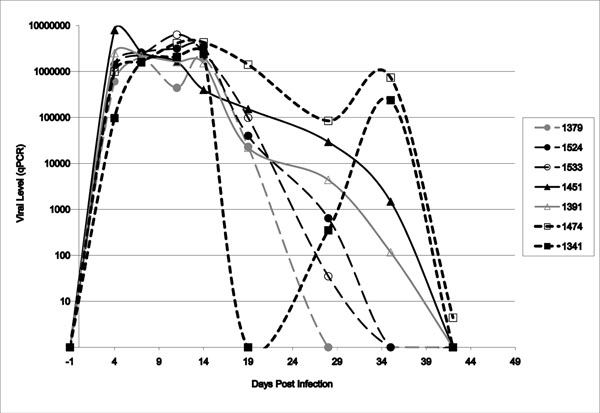
**Different anti-viral reponses associated with PRRS resistance/susceptibility.** PHGC pigs exhibited different anti-PRRSV infection responses as reflected in the serum viral levels after challenge. Circles, solid squares/long dashed lines – resistant pigs (1341, 1379, 1524, 1533); Triangles/solid lines= susceptible pigs (1451, 1391); Squares/short dashed line = pigs with virus reactivation (1341, 1474).

Results have affirmed that all challenged pigs become PRRSV infected with peak viremia from 4-21 dpi (Rowland et al., manuscript in preparation). The PHGC results revealed the appearance of stratified subpopulations of PRRS resistant/ susceptible pigs, which exhibited wide variations in virus load and growth performance; examples of such anti-viral responses are shown in Figure 1. The greatest impact of PRRSV infection was on weight, with only about 30% of infected pigs in the same weight class as the reference control pigs (pigs from the same litters kept uninfected and weighed for the same 42 days). Plotting virus load versus average daily weight gain (ADWG) showed little correlation between growth and virus load. Multivariate statistical analyses of viral load and weight data have categorized PHGC pigs into 4 extreme categories including the most desirable, PRRS resistant low virus/high weight gain (Lv/Hg) pigs, the worst, PRRS susceptible high virus/ low weight gain (Hv/Lg) pigs, the PRRS tolerant, high virus/high weight gain (Hv/Hg) pigs, and the less thrifty, low virus/low weight gain (Lv/Lg) pigs. This statistical categorization of pigs from each PHGC trial provides a critical basis for selecting pigs and samples for detailed analyses of processes that control transcriptional and proteomic responses to PRRSV infection, as outlined in Table 1.

**Table 1:** PRRS resistance/susceptibility traits for genome wide association studies (GWAS) and gene expression pathway analyses

Sample	Trait	Current phenotypic information	Potential phenotypic information
Blood serum [collected at -6, 0, 4, 7, 10, 14, 21, 28, 35, 42 dpi]	Virus levels	PRRS Viral qPCR [4 dpi virus level, 7 dpi virus level, 0-19/21 dpi virus level (= AUC, area under curve); 28 dpi virus level]	PRRSV type 1 and 2 Virus sequence, especially during reactivation Presence of other viruses

Blood serum	Antibody (Ab) levels	IDEXX PRRSV Ab titer	PRRSV Serum Neutralizing Abs Decay of maternal anti-PCV2 Ab Ab to other microbes

Blood serum	Immune Protein levels	Interleukin-8 (IL-8), Interferon-γ (IFN-γ) Selected Luminex bead assays for 8 cytokines	More Luminex bead assays for multiple cytokines, chemokines Broad proteomic analyses

Blood RNA (ABI Tempus tubes for RNA collected at 0, 4, 7, 10, 14, 21, 28, 35, 42 dpi)	Blood gene expression	Microarray (Pigoligoarray) based gene expression QPCR for selected gene expression Identification of “classifier genes” for PRRS resistance	next generation sequencing analyses (RNA-seq) Alternate splicing events Bioinformatic analysis of virus/host correlated gene expression Identify eQTL

Growing pig [during infection]	Weight gain (kg)	Weight gain [Overall 0-42 dpi; Weight gain at peak virus replication]	New trials with growth to market weight and extensive growth and pork quality analyses

Ear tissue	Genomic DNA	SNP genotyping (Illumina PorcineSNP60 Genotyping BeadChip) Targeted SNPs GWAS analyses	Broader SNP analyses More Targeted SNPs, e.g., specific regions or candidate genes More extensive GWAS analyses including eQTL and Ref-seq data

Tonsils [at 42 dpi]	Mucosal tissue; virus reservoir	None	PRRSV levels at 42 dpi - PRRSV persistence Gene expression with persistent virus

Oral fluids [pen samples]	Virus levels	PRRSV levels	PRRSV type 1 and 2 Virus sequence, especially during reactivation Presence of other viruses

Oral fluids	Antibody (Ab) levels	IDEXX PRRSV Ab titer	PRRSV Serum Neutralizing Abs Decay of maternal anti-PCV2 Ab Ab to other microbes Comparison of oral fluid versus serum responses

Oral fluids	Immune Protein levels	Selected Luminex bead assay for cytokines	More Luminex bead assays for multiple cytokines, chemokines Broad proteomic analyses

Sample usage plans for current phenotypic information and projected deeper phenotypic data possible with additional tests and funding.

Sera are now being compared for factors involved in recovery from infection, including speed of anti-viral responses and levels of immune cytokines. Selected whole blood RNA samples are being compared for gene expression using the Pigoligoarrays [19]. In Table 1 are listed the PRRS resistance/susceptibility phenotypic traits currently being tested as well as those that could be collected with additional tests for deeper phenotypic information if additional funding becomes available. This continued phenotyping is possible because of the extensive PHGC planning, detailed sample inventory at KSU and BARC, and coordinated PHGC database. Genomic DNA samples from all trial 1-6 PHGC pigs have already been prepared and genotyped with the Porcine SNP60 SNPchip; a grant submission for the last genotypes and RNA-seq analyses is under review. Genome-wide association studies (GWAS) are now underway to identify alleles and chromosomal regions that are associated with anti-PRRSV infection responses. Overall, the PHGC project will enable researchers to verify important genotypes and phenotypes that predict resistance/susceptibility to PRRSV infection.

## Conclusion

The expected outcomes of the PRRS Host Genetics Consortium are to: 1) Define genomic regions, SNP alleles, or candidate genes [and source pig genetics] which are correlated with PRRS resistance/susceptibility quantitative trait loci (QTL); 2) Use these QTL to develop selection procedures to lower the effects and persistence of PRRSV virus in pigs; 3) Determine why [some] pigs stay healthy despite PRRSV infection; 4) Utilize information gained to help uncover unique PRRSV resistance mechanisms and virus-host interactions, thus highlighting alternate vaccine and therapeutic approaches; and 5) Identify pigs with improved resistance not just to PRRS but to respiratory infections.

## List of abbreviations used

Ab: Antibody; AUC: area under the curve; dpi: days post infection; GWAS: genome-wide association studies; QTL: quantitative trait loci; PCV2: porcine circovirus type 2; PRRSV: porcine reproductive and respiratory syndrome virus; PHGC: PRRS Host Genetics Consortium.

## Competing interests

The authors declare that they have no competing interests.
